# Association of depressive and anxiety symptoms with adverse events in Dutch chronic kidney disease patients: a prospective cohort study

**DOI:** 10.1186/s12882-015-0149-7

**Published:** 2015-09-21

**Authors:** Wim L. Loosman, Marcus A. Rottier, Adriaan Honig, Carl E.H. Siegert

**Affiliations:** Department of Nephrology, Sint Lucas Andreas Hospital, Amsterdam, The Netherlands; Department of Psychiatry, Sint Lucas Andreas Hospital, Amsterdam, The Netherlands; Department of Psychiatry, VU University Medical Center, Amsterdam, The Netherlands

**Keywords:** Depression, Anxiety, Mortality, Hospitalization, Chronic kidney disease

## Abstract

**Background:**

Depressive symptoms have been reported to be associated with adverse clinical outcome in patients with chronic kidney disease (CKD) not on dialysis. This association has not been examined in Europe. Anxiety and depressive symptoms often co-occur. However, as yet there are no data concerning a possible association of anxiety symptoms with adverse clinical outcome. We examined the association of depressive and anxiety symptoms with adverse clinical outcome in Dutch CKD patients not on dialysis.

**Methods:**

In this 3-year follow-up prospective cohort study, CKD patients not on dialysis with an estimated glomerular filtration rate (eGFR) ≤ 35 ml/min/1.73 m^2^ from an urban teaching hospital were selected. Symptoms of depression and anxiety were evaluated using the Beck Depression Inventory (BDI) and the Beck Anxiety Inventory (BAI). Cox proportional hazards models were used to calculate hazard ratio’s (HRs) with a composite event of death, initiation of dialysis, and hospitalization as outcome. HRs were adjusted for age, gender, diabetes, cardiovascular disease and eGFR.

**Results:**

Of 100 included CKD patients depressive and anxiety symptoms were present in 34 and 31 %, respectively. Adjusted HRs for the composite event for patients with depressive and anxiety symptoms were 2.0 (95 % confidence interval (CI) 1.2–3.5) and 1.6 (95 % CI 0.9–2.7), respectively. Twenty three patients had both depressive and anxiety symptoms of whom adjusted HRs were 2.2 (95 % CI 1.2–4.0) for a composite event.

**Conclusions:**

Depressive and anxiety symptoms are common in patients with CKD in The Netherlands. Depressive symptoms are associated with an increased risk of poor clinical outcome. Anxiety symptoms show a trend for an increased risk of poor clinical outcome. There seems to be no additive effect of anxiety symptoms in addition to depressive symptoms with regard to poor clinical outcome.

## Background

Chronic kidney disease (CKD) is a highly prevalent disorder that affects around 10 percent of the global population [1]. The prevalence and incidence of renal replacement therapy (RRT) has increased dramatically and is predicted to further increase 29 % and 47 % respectively until 2020 [2]. Despite traditional risk factors for initiation of dialysis, such as cardiovascular disease, proteinuria, and hyperglycaemia [3, 4], there is an increasing interest in psychosocial risk factors [5, 6], such as depressive symptoms [7].

Depressive symptoms are common in patients with end-stage renal disease (ESRD) [8] and associated with mortality [9, 10]. In patients with earlier-stage CKD (stage 2–5) the prevalence of depressive symptoms is high, ranging from 7 % to 42 % [11–13] and is also associated with adverse clinical outcomes such as hospitalization, initiation of dialysis, or death [7, 11, 12, 14, 15]. This association has only been investigated in US and Asian cohorts and not in Europe. There are important distinctions between study cohorts of CKD patients in different continents. First, the ESRD incidence is lower in in Europe, compared to the US and Asia [16–18]. In addition, the relative risk for progression from CKD stages 3 or 4 to ESRD in US white patients compared with Norwegian patients was found to be 2.5, despite a similar CKD prevalence [19]. Therefore, risk factors for initiation of dialysis could vary in different continents. Secondly, demographic differences between Europe and the US may lead to differences in progression to ESRD [20]. Thirdly, there are differences in the health care system between the US and Europe [20, 21].

Anxiety symptoms often co-occur with depressive symptoms and are, in addition to depressive symptoms, also of interest in patients with ESRD [22]. In this patient group anxiety symptoms are associated with impaired health related quality of life (HRQOL), and even seem to aggravate the relationship between depressive symptoms and impaired HRQOL [22] . The prevalence of anxiety symptoms varies between 13 and 50 % [22–24] in ESRD patients but the prevalence of anxiety symptoms remains unknown in patients with earlier-stage CKD (stage 2–5). Furthermore, it is unknown whether anxiety symptoms are associated with adverse clinical outcome such as death, initiation of dialysis or hospitalization.

The aim of this study is to evaluate the prevalence of depressive and anxiety symptoms in Dutch CKD patients, and to examine the association of these symptoms with adverse clinical outcome, defined as death, initiation of dialysis or hospitalization. A secondary aim is to assess the additional effect of anxiety symptoms on the association between depressive symptoms and adverse clinical outcome.

## Method

### Patients characteristics

All prevalent CKD patients not on dialysis in an urban primary care hospital, the Sint Lucas Andreas Hospital in Amsterdam the Netherlands, aged ≥18 years with an estimated glomerular filtration rate (eGFR) of ≤ 35 ml/min/1.73 m^2^ were eligible for the study. The eGFR was calculated using the Modification of Diet in Renal Disease (MDRD) equation [25]. Recruitment ran in the outpatients clinic over a 3-month period from September 2011 until December 2011 and was complete when the first hundred patients were included. Patients were followed until 12 December 2014. Patients unable to complete the Dutch, English, or Turkish translation of the questionnaires, and patients who were otherwise unable to complete the questionnaires were excluded. The study was approved by the Medical Ethical Committee (MEC) of the Sint Lucas Andreas Hospital (reference number of the approval: mec11/115 IDO). Informed consent was obtained from all patients prior to enrolment.

### Psychiatric assessment

Depressive symptoms were measured using the Beck Depression Inventory (BDI) [26] and symptoms of anxiety were measured using the Beck Anxiety Inventory (BAI) [27]. Both questionnaires consist of 21 questions which are scored on a 0 – 3 scale. Patients were considered to have depressive symptoms when they scored ≥11 points on the BDI, with a reported sensitivity and specificity of 88 and 89 %, respectively [28]. Patients were considered to have anxiety symptoms when they scored ≥ 13 points on the BAI with a reported sensitivity and specificity of 76 and 81 %, respectively [29, 30].

### Demographic and clinical characteristics

Socio demographic, clinical and laboratory characteristics were all identified from electronic medical records. Socio demographic characteristics included age, gender, ethnicity, marital status having children and working status. Clinical characteristics included smoking status, psychiatric history, present prescription of antidepressants, diabetes mellitus (DM) and cardiovascular disease (previous myocardial infarction (MI), heart failure and known coronary artery disease.

### Clinical outcomes

The primary outcome was an event defined as a composite of death and initiation of dialysis treatment, or the previous two events combined with first hospitalization [7]. Secondary outcomes were the occurrence of each of these 3 events assessed separately during 3-year follow-up.

In addition eGFR was obtained from medical records before death, initiation of dialysis, or kidney transplantation. To determine the decrease in kidney function, two eGFR values were used: one value at baseline and one at time of follow up or just before death, initiation of dialysis, or kidney transplantation. Decrease in kidney function was defined as eGFR decrease in millimetres per minute per 1.73 m^2^ (ml/min/1.73 m^2^).

### Statistical analysis

Descriptive statistics and patient data listings were used to summarize the data collected on the Case Report File. Continuous variables were summarized using means and standard deviations (SD). Categorical variables were described using frequencies and percentages. Continuous variables were compared with the use of Student’s t-test or the Mann–Whitney U-test for skewed data. Comparison between groups for categorical variables was performed using the chi-square of Fisher exact test when appropriate.

Median survival time was measured using a Kaplan Meier and median follow up with reverse Kaplan Meier methods [31]. Patients who had a kidney transplantation, were lost to follow up or reached the end of the study period at December 2014 were censored. Incidence rates of adverse events were calculated per 1000 person-months. Cox proportional hazard models were used to estimate unadjusted and adjusted hazard ratios (HRs) of a composite event (initiation of dialysis, death and first hospitalization), a combination of death and dialysis, or each event separately. These models were conducted for BDI and BAI scores as dichotomized variable (presence versus absence of depressive or anxiety symptoms) and for BDI and BAI score as a continuous variable. Assumptions of the proportional hazard model were confirmed by using cumulative hazard plots. Furthermore, to determine if there is an additional effect of anxiety symptoms on the association of depressive symptoms and adverse events, depressive and anxiety symptoms patients were categorized into four groups: neither depressive or anxiety symptoms, both depressive and anxiety symptoms, only depressive symptoms and only anxiety symptoms. HRs were calculated for each group with neither depressive or anxiety symptoms as a reference group and all HRs are presented with 95 % confidence intervals (CI). Age, gender, diabetes, cardiovascular disease and eGFR were entered in the adjusted model as covariates. These variables were based on previous reports on depressive symptoms and mortality among patients with CKD. Before conducting multivariable analyses we used the variance inflation factor (VIF) in order to control for possible multicollinearity effects. Decrease in kidney function between low and high depressive symptoms were analysed with a Student’s t-test. A P-value of < 0.05 (two-sided) was used to indicate statistical significance. Statistical analysis was performed with SPSS version 18.0.

## Results

### Patients characteristics

During the inclusion period, 160 CKD patients were approached of whom 60 patients were excluded because of motivational reasons, language barriers, or patients were otherwise unable to fill out the questionnaires. Baseline characteristics of 100 CKD patients with and without depressive or anxiety symptoms are shown in Table 1. Thirty-four patients (34 %) had depressive symptoms, defined as a BDI score ≥ 11. Patients with depressive symptoms had more often a history of depression (*p* = 0.03) and cardiovascular disease (*p* = 0.02). Thirty-one patients (31 %) had anxiety symptoms, defined as a BAI score ≥ 13, of whom more female patients (*p* = 0.04). There were no further differences at baseline between patients with and without depressive or anxiety symptoms (Table 1).

**Table 1:** Baseline characteristics of 100 CKD patients with and without depressive or anxiety symptoms

	All patients	*BDI < 11*	*BDI ≥ 11*	P-value	*BAI < 13*	*BAI ≥ 13*	*P*-value
	(*n* = 100)	(*n* = 66)	(*n* = 34)		(*n* = 69)	(*n* = 31)	
Socio demographic characteristics							
Age, years	67.9 (14.5)	67.8 (13.3)	67.8 (16.8)	*P* = 0.99	68.1 (12.9)	67.3 (17.7)	*P* = 0.81
Sex, % male	57	62	47	*P* = 0.15	64	42	*P* = 0.04
Ethnicity, % Dutch	82	83	79	*P* = 0.63	78	90	*P* = 0.15
Married or living together, % yes	58	53	71	*P* = 0.09	63	52	*P* = 0.30
Children, % yes	74	79	81	*P* = 0.77	78	83	*P* = 0.53
Employed, % yes	16	19	13	*P* = 0.44	18	13	*P* = 0.56
Clinical parameters							
BMI, kg/m^2^	28.6 (5.5)	28.2 (5.2)	29.3 (6.1)	*P* = 0.35	28.2 (5.3)	29.5 (5.8)	*P* = 0.30
Current smoking, % yes	24	28	24	*P* = 0.69	25	30	*P* = 0.61
Comorbidity, % yes							
Diabetes Mellitus	36	36	35	*P* = 0.92	35	39	*P* = 0.71
Hypercholesterolemia	36	38	32	*P* = 0.57	39	29	*P* = 0.33
Hypertension	76	80	68	*P* = 0.16	77	74	*P* = 0.78
Cardiovascular disease	40	32	56	*P* = 0.02	35	52	*P* = 0.11
Peripheral vascular disease	7	6	9	*P* = 0.61	9	3	*P* = 0.32
Cerebrovascular disease	14	14	15	*P* = 0.88	15	13	*P* = 0.83
COPD	9	9	9	*P* = 0.97	7	13	*P* = 0.36
History of non-skin cancer	19	18	21	*P* = 0.77	20	16	*P* = 0.62
Previous depression	7	3	15	*P* = 0.03	4	13	*P* = 0.12
Use of antidepressants, % yes	7	5	12	*P* = 0.18	7	7	*P* = 0.89
Laboratory values							
Hemoglobin, g/l	7.5 (0.9)	7.5 (0.7)	7.3 (1.1)	*P* = 0.21	7.5 (0.7)	7.4 (1.1)	*P* = 0.51
Phophorus, mmol/l	1.35 (0.25)	1.32 (0.23)	1.41 (0.30)	*P* = 0.14	1.34 (0.24)	1.38 (0.29)	*P* = 0.56
Calcium, mmol/l	2.32 (0.15)	2.32 (0.16)	2.32 (0.14)	*P* = 0.87	2.32 (0.16)	2.32 (0.14)	*P* = 0.93
Albumin, g/l	39.9 (4.6)	39.9 (3.4)	39.9 (5.1)	*P* = 0.99	40.2 (4.9)	38.9 (3.5)	*P* = 0.34
Creatinine, umol/l	269 (101)	263 (80)	282 (135)	*P* = 0.39	273 (96)	262 (114)	*P* = 0.62
eGFR, ml/min/1,73 m^2^	20.4 (6.3)	20.8 (6.2)	19.8 (6.6)	*P* = 0.49	20.3 (6.4)	20.7 (6.4)	*P* = 0.78

Values expressed as percentage for categorical variables and mean ± standard deviation or mean (range), as appropriate

Abbreviations: COPD: chronic obstructive pulmonary disease, eGFR: estimated glomerular filtration rate

### Follow up and outcomes

Median follow up was 3.1 years (minimum of 0.6, maximum 3.2).Seven patients lost to follow up, of whom one patient changed to another hospital and six patients did not show up at the outpatient clinic. Three patients had a kidney transplantation during follow up. In total, 74 patients had a composite event (death, initiation of dialysis, or hospitalization) of whom 43 patients died or were on dialysis. Differences between patients with and without depressive and anxiety symptoms are presented in Table 2. In patients with and without depressive symptoms, respectively 33 and 50 % of deaths, and 16 and 17 % of hospitalizations were due to cardiovascular disease.

**Table 2:** Association of depressive/anxiety symptoms with death, dialysis, and hospitalization in CKD patients

	*Events*		*Incidence* ^a^			
	*BDI < 11*	*BDI ≥ 11*	*BDI < 11*	*BDI ≥ 11*	*BDI ≥ 11*	*BDI ≥ 11*
	(n)	(n)			Unadjusted model	Adjusted model ^b^
Dialysis/Death	24	19	13.2	25.8	1.9 (1.0 – 3.5)*	2.1 (1.0 – 4.2)*
Composite event ^c^	44	30	35.7	81.7	2.0 (1.3 – 3.3)	2.0 (1.2 – 3.5)
Death	10	9	5.5	12.2	2.2 (0.9 – 5.4)	1.5 (0.5 – 4.5)
Dialysis	14	10	7.7	13.6	1.7 (0.7 – 3.8)	2.1 (0.8 – 5.4)
Hospitalization	36	25	29.2	68.1	2.0 (1.2 – 3.4)	1.9 (1.1 – 3.5)
	Events		Incidence ^a^			
	*BAI < 13*	*BAI ≥ 13*	*BAI < 13*	*BAI ≥ 13*	*BAI ≥ 13*	*BAI ≥ 13*
	(n)	(n)			Unadjusted model	Adjusted model ^b^
Dialysis/Death	28	15	15.1	21.6	1.3 (0.7 – 2.5)	1.6 (0.8 – 3.2)
Composite event ^c^	48	26	38.5	73.8	1.7 (1.0 – 2.8)*	1.6 (0.9 – 2.7)
Death	11	8	5.9	11.5	1.6 (0.6 – 4.0)	1.5 (0.5 – 4.2)
Dialysis	17	7	9.2	10.1	1.1 (0.5 – 2.6)	1.4 (0.5 – 3.7)
Hospitalization	39	22	21.0	31.7	1.7 (0.9 – 2.9)	1.6 (0.9 – 2.8)

Values expressed as hazard ratio (95 % confidence interval)

*P < 0.05

^a^incidence rate of adverse events presented per 1000 person months

^b^Adjusted for age, gender, history of depression, diabetes, cardiovascular disease and glomerular filtration rate

^c^Composite event: death, initiation of dialysis or first hospitalization

### Survival

**Fig. 1:**
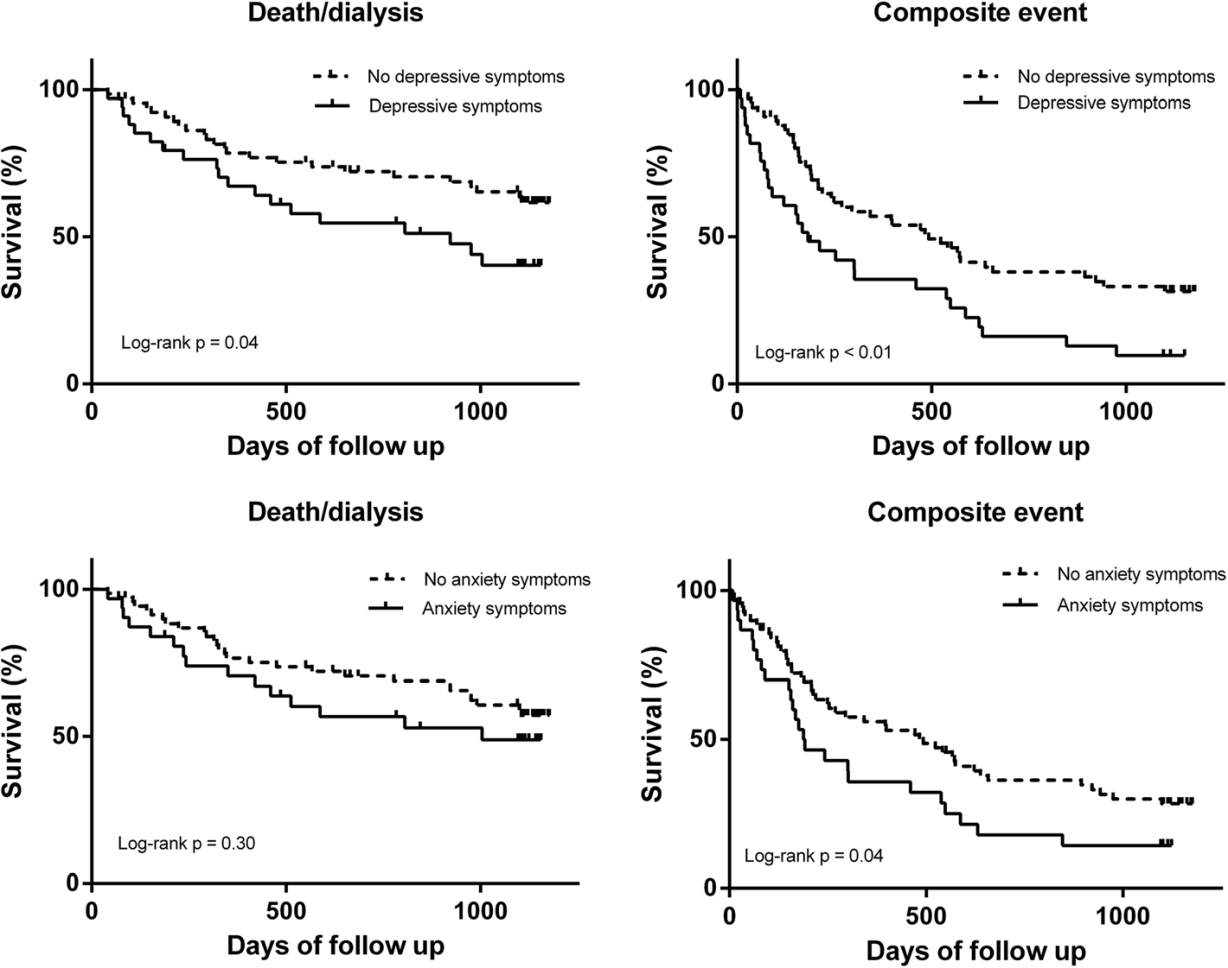
Kaplan-Meier survival curves for CKD patients with and without depressive or anxiety symptoms. Outcome is defined as death/dialysis or a composite event (death, dialysis or first hospitalisation)

The survival curves for the presence of depressive and anxiety symptoms during 3 years of follow up showed a higher cumulative incidence of death/dialysis or a composite event for patients with depressive or anxiety symptoms (Fig. 1). Unadjusted and adjusted (adjusted for age, gender, history of depression, diabetes, cardiovascular disease and eGFR) HRs for patients with depressive and anxiety symptoms are presented in Table 2. Patients with depressive symptoms had an adjusted HR of 2.1 (95%CI: 1.0 – 4.2) and 2.0 (95%CI: 1.2 – 3.5) for death/dialysis and a composite event, respectively. Patients with anxiety symptoms had an adjusted HR of 1.6 (95%CI: 0.8 – 3.2) and 1.6 (95%CI: 0.9 – 2.7) for death/dialysis and a composite event, respectively. Unadjusted and adjusted HRs for each unit increase of the BDI and BAI are presented in Table 3. Per unit increase of the BDI adjusted HRs were 1.07 (95%CI: 1.01 – 1.13) and 1.06 (95%CI: 1.02 – 1.11) for death/dialysis and a composite event, respectively. Per unit increase of the BAI adjusted HRs were 1.04 (95%CI: 1.00 – 1.09) and 1.05 (95%CI: 1.02 – 1.09) for death/dialysis and a composite event, respectively.

**Table 3:** Association of depressive/anxiety symptoms (per unit increase) with death, dialysis, and hospitalisation in CKD patients

	*BDI/unit increase*	*BDI/unit increase*	*BAI/unit increase*	*BAI/unit increase*
	Unadjusted	Adjusted ^a^	Unadjusted	Adjusted ^a^
Dialysis/Death	1.05 (1.01 – 1.10)	1.07 (1.01 – 1.13)	1.03 (0.99 – 1.07)	1.04 (1.00 – 1.09)*
Composite event ^b^	1.05 (1.02 – 1.09)	1.06 (1.02 – 1.11)	1.04 (1.01 – 1.08)	1.05 (1.02 – 1.09)
Death	1.07 (0.99 – 1.14)	1.04 (0.97 – 1.13)	1.03 (0.97 – 1.10)	1.01 (0.95 – 1.09)
Dialysis	1.04 (0.98 – 1.12)	1.08 (1.01 – 1.16)	1.02 (0.97 – 1.08)	1.05 (0.99 – 1.11)
Hospitalisation	1.05 (1.01 – 1.09)	1.06 (1.01 – 1.10)	1.04 (1.01 – 1.09)	1.05 (1.02 – 1.09)

Values expressed as hazard ratio (95 % confidence interval)

*P < 0.05

^a^Adjusted for age, gender, history of depression, diabetes, cardiovascular disease and glomerular filtration rate

^b^Composite event: death, initiation of dialysis or first hospitalisation

Fifty eight patients had neither depressive or anxiety symptoms, 23 had both depressive or anxiety symptoms, 11 had only depressive symptoms and 8 had only anxiety symptoms. HRs for these four categories are presented in Table 4. Patients with both depression and anxiety had an adjusted HR of 2.2 (95%CI: 1.0 – 4.8) and 2.2 (95%CI: 1.2 – 4.0) for death/dialysis or a composite event, respectively.

**Table 4:** Association of depressive/anxiety categories with death, dialysis, and hospitalization in CKD patients

	Neither depression or anxiety	Both depression and anxiety	Only depression	Only anxiety
	(*N* = 58)	(*N* = 23)	(*N* = 11)	(*N* = 8)
*Dialysis/death* Unadjusted	Reference	1.9 (0.9 – 3.9)	1.5 (0.6 – 3.8)	0.6 (0.1 – 2.6)
*Dialysis/death* Adjusted ^a^	Reference	2.2 (1.0 – 4.8)*	1.8 (0.6 – 4.8)	0.9 (0.2 – 4.2)
*Composite event* ^*b*^ Unadjusted	Reference	2.1 (1.2 – 3.7)	2.2 (1.1 – 4.4)	1.5 (0.6 – 3.6)
*Composite event* ^*b*^ Adjusted ^a^	Reference	2.2 (1.2 – 4.0)	2.3 (1.0 – 5.3)*	1.6 (0.6 – 4.1)

Values expressed as hazard ratio (95 % confidence interval)

*P < 0.05

^a^Adjusted for age, gender, history of depression, diabetes, cardiovascular disease and glomerular filtration rate

^b^Composite event: death, initiation of dialysis or first hospitalization

### Kidney function

During 3-year follow up, the decrease in kidney function was 1.9 (SD 8.5) and 2.2 (SD 8.7) ml/min/1.73 m^2^ (*p* = 0.91) for patients with and without depressive symptoms, respectively. The decrease in kidney function was 2.8 (SD 7.2) and 1.9 (SD 9.2) ml/min/1.73 m^2^ (*p* = 0.66) for patients with and without anxiety symptoms, respectively.

## Discussion

This 3-year prospective single centre study in a cohort of Dutch CKD patients examined the association between depressive and anxiety symptoms with the incidence of adverse events defined as death, initiation of dialysis, and hospitalization or a combination of these events. Patients with depressive symptoms appear to have a higher risk of progression to adverse events. Patients with anxiety symptoms showed a trend of progression to adverse events, and did not seem to contribute to the association between depressive symptoms and adverse events.

Our study showed that patients with depressive symptoms have a twofold higher risk of progression to death/dialysis and a composite event (death, initiation of dialysis or hospitalization). These results are comparable to previous reported studies [7, 11, 12, 15] from primarily US CKD cohorts. For example, Hedayati et al. [7]. found that depression at baseline predicts progression to a composite event of death, dialysis, or hospitalization. Despite the differences in health care systems, lower incidence of ESRD in Europe [16, 17, 19], and differences in patient characteristics between European patients and patients from US, the association between depressive symptoms and adverse clinical outcome seems universally present in patients with CKD. Furthermore, our findings demonstrate that the presence of depressive symptoms does not influence a decrease of kidney function. This finding is in line with a prior study of individuals 65 years and older (Cardiovascular Health Study) [32], but not with two recent studies that did establish a positive correlation between depressive symptoms and a faster decrease in eGFR or progression to dialysis [7, 12]. Difference in time of follow up, sample size or diagnostic tools to identify depressive symptoms are possible explanations for this discrepancy.

The presence of anxiety symptoms seem to have a trend of progression to adverse events and did not seem to contribute to the association between depressive symptoms and adverse events. This is the first study to examine this association in CKD patients not on dialysis. In patients with ESRD disease, however, Preljevic et al. [22] showed that there was no additional effect of anxiety symptoms on the relationship of depressive symptoms with mortality. Although, depressive and anxiety symptoms separately also did not have an effect on mortality. Furthermore, our results are comparable to a study in patients with cardiac disease [33]. The authors concluded that anxiety was associated with all-cause mortality, but has no additional value in case of co-occurring depression [33]. Further research in a larger cohort should evaluate the effect of anxiety symptoms on adverse clinical outcome in patients with CKD not on dialysis.

The potential mechanisms responsible for the association of depressive symptoms with adverse clinical outcomes are not completely clear. One plausible mechanism is non-adherence to medical treatment. This phenomenon was demonstrated in hemodialysis patients with depressive symptoms who were demonstrated to be less compliant to follow dietary and fluid restrictions or other recommendations [34]. Another potential mechanism could be that depression is associated with the activation of the hypothalamic-pituitary-adrenal (HPA) axis and sympatho-adrenal hyperactivity, leading to an increased release of cortisol and catecholamine’s respectively. This may lead to immune dysfunction, coagulation abnormalities, platelet and vascular endothelial dysfunctions, which is linked to increased inflammation and cardiovascular events [35–37]. A third possibility is that high depressive symptoms are merely a surrogate marker for comorbidity such as cardiovascular diseases [38]. However, in our study we found a higher prevalence of cardiovascular diseases in the patients with depressive symptoms, but after adjusting for cardiovascular disease, the association between depressive symptoms and poor outcomes remained the same.

This study has some limitations. First, study participants were included from a single centre with a GFR of ≤ 35 ml. Excluded patients were not studied in a similar way as included patients. Therefore, our results may not be applicable to other patients with other CKD stages. Nevertheless, Hedayati *et al.* [7]. found a comparable prevalence of depression in pre-dialysis patients with lower CKD stages Secondly, the BDI cut-off value of 11 has been validated in pre-dialysis CKD patients [28] whereas the BAI cut-off value of 13, which was used in dialysis patients before [29], was validated in a general population [30] . Therefore, the prevalence of depressive and anxiety symptoms can differ based on the chosen cut-off point. We do not think this influences our conclusion because the reported associations with adverse events remained stable after using units increment of both questionnaires. Third, we only assessed depressive and anxiety symptoms at baseline, whereas depressive and anxiety symptoms may change over time. However, this will probably not influence our conclusion, because by not using time-dependent variables the association is most likely underestimated rather than overestimated [39]. Future research could focus on the short term effect of depressive and anxiety symptoms by using multiple measurements of depressive and anxiety symptoms.

## Conclusion

Depressive and anxiety symptoms are common in patients with CKD in The Netherlands. Depressive symptoms are associated with an increased risk of poor clinical outcome. Anxiety symptoms show a trend for an increased risk of poor clinical outcome. There seems to be no cumulative effect of anxiety symptoms in addition to depressive symptoms. Depression and anxiety symptoms should be evaluated early and future research should address appropriate therapeutic regimens and evaluate the effect of treatment of depression on clinical outcome.

## Abbreviations

eGFR: estimated glomerular filtration rate
CKD: Chronic kidney disease
ESRD: End-stage renal disease
HR: Hazard ratio
BDI: Beck depression inventory
BAI: Beck anxiety inventory
CI: Confidence interval
SD: Standard deviation
HRQOL: Health related quality of life
